# Correlation between *CTLA-4* and *CD40* gene polymorphisms and their interaction in graves’ disease in a Chinese Han population

**DOI:** 10.1186/s12881-018-0665-y

**Published:** 2018-09-17

**Authors:** Xiaoming Chen, Zhuoqing Hu, Meilian Liu, Huaqian Li, Chanbo Liang, Wei Li, Liwen Bao, Manyang Chen, Ge Wu

**Affiliations:** Department of Endocrinology, Affiliated Hospital of Guangdong Medical University, Zhanjiang, 524001 China

**Keywords:** Graves’ disease, CD40, CTLA-4, Genetic polymorphism

## Abstract

**Background:**

Single-nucleotide polymorphism (SNP) haplotype and SNP-SNP interactions of *CTLA-4* and *CD40* genes, with susceptibility to Graves’ disease (GD), were explored in a Chinese Han population.

**Methods:**

SNP were genotyped by high resolution melting (HRM). Use the method of Pearson *χ2* test and Logistic regression for the association between single SNP and Graves’ disease. Using the method of *χ2* test and Multifactor Dimensionality Reduction (MDR) to analysis the haplotype frequency distribution, the interaction of SNPs respectively.

**Results:**

Genotypic and allelic frequencies of SNP rs231775, rs3087243 and rs1883832 were statistically different between controls and GD (*p* < 0.05). Mutant allelic frequency of G rs231775 was higher, and A and T allelic frequencies of rs3087243 and rs1883832 were lower in GD than in controls (*P* < 0.05). In *CTLA-4* rs1024161, rs5742909, rs231775, rs231777, rs231779, rs3087243 and rs11571319 showed *D’* < 50% and *r*^*2*^ < 0.3 among each SNP. We identified six commonly found haplotypes; TCGCTGC was associated with the highest GD risk (*OR* = 2.565) and TCACTAC the lowest (*OR* = 0.096). MDR analysis indicated interactions among the rs231775 GG, rs231779 TT and rs3087243 GG genotypes in *CTLA-4* might increase GD risk by 2.53-fold (*OR* = 2.53).

**Conclusion:**

*CTLA-4* and *CD40* were associated with GD incidence in a Chinese Han population. The TCGCTGC and TCACTAC haplotypes in the CTLA-4 gene, were risk and protective factors for Graves’disease respectively. Interactions among the SNPs of rs231775, rs231779 and rs3087243 significantly increase the susceptibility to GD.

## Background

As a thyroid-specific autoimmune disease, Graves’ disease (GD) is the most common cause of hyperthyroidism. The pathogenesis of GD is unclear; however, genome-wide association studies (GWAS) indicate that the genetic background of GD is decided by several genes with differential penetrance. Moreover, GD is a complex disease that is associated with gene-gene and gene-environment interactions [1, 2]. Interactions between costimulatory molecules and downstream cytokines (such as IL-2) that are regulated by CD28 (CTLA-4)/B7 and CD40/CD40L are important mechanisms that constitute the immune response. Studies show that abnormalities of these common signaling pathways are associated with multiple autoimmune diseases (AID) such as diabetes, scleroderma and autoimmune thyroid disease [3]. Correlations between *CTLA-4* and GD susceptibility are complex, wherein mutations play a significant role, and specific sites in those mutations are both unstable and correlated with GD in different populations [4–7]. For instance, the +49A/G polymorphism in exon 1 results in a threonine-to-alanine conversion, which Gu et al. showed to be associated with GD in the Chinese Han population [5]. By contrast, others found that the +49A/G polymorphism in exon 1 was irrelevant in the Japanese, Brazil and Lebanese populations [4, 6, 7]. Linkage analysis, candidate gene analysis and GWAS unanimously confirmed that the CD40–1 C/T polymorphism located at 20q11.2-20q13 was stably associated with GD susceptibility [8–10]. The Kozak consensus sequence is a necessary nucleotide fragment in the initiation of translation of CD40, while the CC genotype and allele C can both increase mRNA translation efficiency of the CD40 gene and thus increase its expression by 15–32% [11, 12].

There are very many genetic mutations and sophisticated haplotype models in the *CTLA-4* and *CD40* genes, whose correlation with particular disease states are far more complex than had originally been appreciated. A recent study showed that there may be a combined and additive effect between the CD40–1C > T and CTLA-4-6230G > A polymorphisms with the development of GD [13]. Thus, we have combined multiple known polymorphic loci before performing correlation analyses between the haplotypes and GD. The over-arching objective of this approach was to provide a more accurate genetic exploration of the susceptibility of GD. In addition, we present an in-depth analysis of the correlation between *CTLA-4* and *CD40* genes against GD susceptibility via interaction analysis of *CTLA-4* and *CD40* in cases of GD as compared with a control population.

## Methods

### Subjects and grouping

This was a retrospective analysis of data that were collected from 260 GD patients (48 males and 212 females, with a mean age of 36.2 ± 15.2 years) from the outpatient or inpatient departments of Endocrinology of the Affiliated Hospital of Guangdong Medical University between June 2013 and June 2014 (specific data are shown in Table 1).

**Table 1:** Basic clinical characteristics of the subjects

	GD group	Control	*p* value
Sex (male/female)	48/212	104/144	0.001
Age (years)	36.2 ± 15.2	37.4 ± 11.3	0.263
FT3 (pmol/L)	13.28 ± 13.12	4.38 ± 3.61	< 0.0001
FT4 (pmol/L)	31.53 ± 28.10	16.33 ± 3.61	< 0.0001
TSH (mUL/L)	3.02 ± 8.72	2.07 ± 1.11	< 0.0001
TRAb (IU/L)	13.55 ± 14.01	0.51 ± 0.50	< 0.0001

*Note:* Variants are expressed as mean ± standard deviation (M ± SD) or N; *FT3* free T3; *FT4* free T4; *TSH* thyroid-stimulating hormone; *TRAb* TSH-receptor antibody; *GD* Graves’ Disease

The inclusion criteria for the patients with GD were based upon the clinical diagnostic criteria of GD in the Internal Medicine manual (8th edition, People’s Medical publishing house) as follows: (1) The patients met the clinical diagnostic criteria of GD. (2) Patients without other serious diseases, such as hypertension, malignancy, chronic liver and kidney diseases. (3) The subjects and other immediate family members over three generations had no autoimmune diseases except GD. (4) Three generations of the patients’ family were of Han nationality and were resident in Western Guangdong.

Over the same period, 248 healthy volunteers (104 males and 144 females, with a mean age of 37.4 ± 11.3 years) were selected as the normal control group. The healthy control group consisted of healthy individuals who underwent physical examination in the Affiliated Hospital of Guangdong Medical University during the same period. The inclusion criteria for the healthy control group were as follows: (1) There was no abnormality in medical history, physical examination, blood glucose examination, blood pressure, blood lipids and other biochemical tests through inquiry. (2) The subjects and other immediate family members over three generations had no autoimmune diseases including GD.

The exclusion criteria in both the GD case group and the healthy control group were as follows: (1) Inability to extract enough DNA to perform the classification experiment due to coagulation in serum sample. For example, improper storage of blood samples, improper transportation of blood samples as well as anticoagulation tube-induced coagulation. (2) There was a serious lack of information or serious information bias for the subject scale. (3) GD patients, controls, or other immediate family members in three generations had other autoimmune diseases except for GD.

Ethical approval was obtained from the Medical Ethics Committee of the Affiliated Hospital of Guangdong Medical University. The approval number was: PJ2012029. All the above enrolled patients signed an informed consent document.

### SNP genotyping

The *CTLA-4* SNPs (with a minor allele frequency ≤ 5%, to exclude rare mutations from the analysis) covering the exons, introns, 5’-UTR, and 3’-UTR were selected. The SNP rs1024161 in the non-encoding area between *CTLA-4* and *KRT18P39* genes, and the *CD40*–1C/T polymorphic loci (rs1883832) were also selected. The PCR-HRM curve was used to genotype SNPs, adopting a 10-μl PCR reaction assay system containing 5 μl Premix Taq™, 0.2 μM of each primer, 50 ng DNA, and sterile water. The following reaction conditions were applied: 3 min denaturation at 94 °C, followed by 30 cycles of a denaturation step (30 s at 94 °C), an annealing step (30 s at the Tm annealing temperature), and an elongation step (30 s at 72 °C), and a final elongation reaction at 72 °C for 10 min. Table 2 lists the PCR primers and Tm values of each SNP. Following the manufacturer’s instructions, a Lightscanner 96 (LS96, Idaho Technology Inc.) was used to scan the reacted 96-well plate and record the data. The HRM-High and HRM-Low Tm internal calibrations were synthesized by Sangon Biotech (Shanghai, China). The sequences were as follows:

**Table 2:** SNP PCR primers

SNP	Primer	Size (bp)	Tm (°C)
rs1024161	F:AGAAATTTGAGTTAAAGGCTCT R:GAATGTACAGATAATGTCACTCT	102	53
rs5742909	F:CTCCAAGTCTCCACTTAGTT R:GAAGCTTCATGTTCACTTT	51	53
rs231775	F:CCTTGGATTTCAGCGGCAC R:AGAGTGCAGGGCCAGGT	63	59.7
rs231777	F:ATTGAATCTCAACCTTATCTCTCTC R:ACCTACTTCATACAAACTACATGG	70	56.2
rs231779	F:TGCAGCCACTATTTTTGAGTTGA R:ACACTCCCATGCTCCTTTGT	96	59.2
rs3087243	F:TTCACCACTATTTGGGATATAA R:GTGTTAAACAGCATGCCAAT	80	53
rs11571319	F:TGGGTTAACACAGACATA R:CCTGTGTTAAACAGCATGCCA	59	50.5
rs11571302	F:ATGGGTTGTTCCACGACTTC R:AAACGCTGCCAATAAACAGTC	76	57
rs11571297	F:TTACTTTTAACTTCCATTCCCAGC R:TCTACCAGAAGTTGAAGTGTAGG	82	57.3
rs1883832	F:CTGCCGCCTGGTCTCAC R:ACTGCAGAGGCAGACGAAC	45	60

HRM-High Tm internal calibration sequence:

GCGGTCAGTCGGCCTAGCGGTAGCCAGCTGCGGCACTGCGTGACGCTCAG.

HRM-Low Tm internal calibration sequence:

CTGAGCGTCACGCAGTGCCGCAGCTGGCTACCGCTAGGCCGACTGACCGC.

### Statistical analysis

Quantitative data were expressed as mean ± standard deviation (M ± SD), while qualitative data were expressed as frequency or percentage. When the data were not normally distributed, they were compared after being converted by the appropriate conversion functions. Independent samples between two groups were analyzed by the Student’s T-test, while comparisons of independent samples among three or more groups were assessed by analysis of variance using SPSS 22.0 (IBM Corp., NY, USA). The Pearson’s goodness-of-fit test, i.e., the Hardy-Weinberg equilibrium equation was adopted. The robust χ2 test was first used to evaluate the correlation between single SNP and GD, and then a common gene model was adopted. Unconditional logistical regression analysis was used to explore the correlation between the SNP allele/genotype and GD. The SHEs is an online software program (http://analysis.bio-x.cn/myAnalysis.php) that was originally used to analyze the leakage of the common *CTLA-4* SNPs in the control group. Pearson’s χ2 test was then used to conduct haplotypic analysis between case and control groups. Multifactor dimensionality reduction (MDR) analysis software was adopted to analyze SNP-SNP interactions.

## Results

### Results of the HRM genotyping

**Fig. 1:**
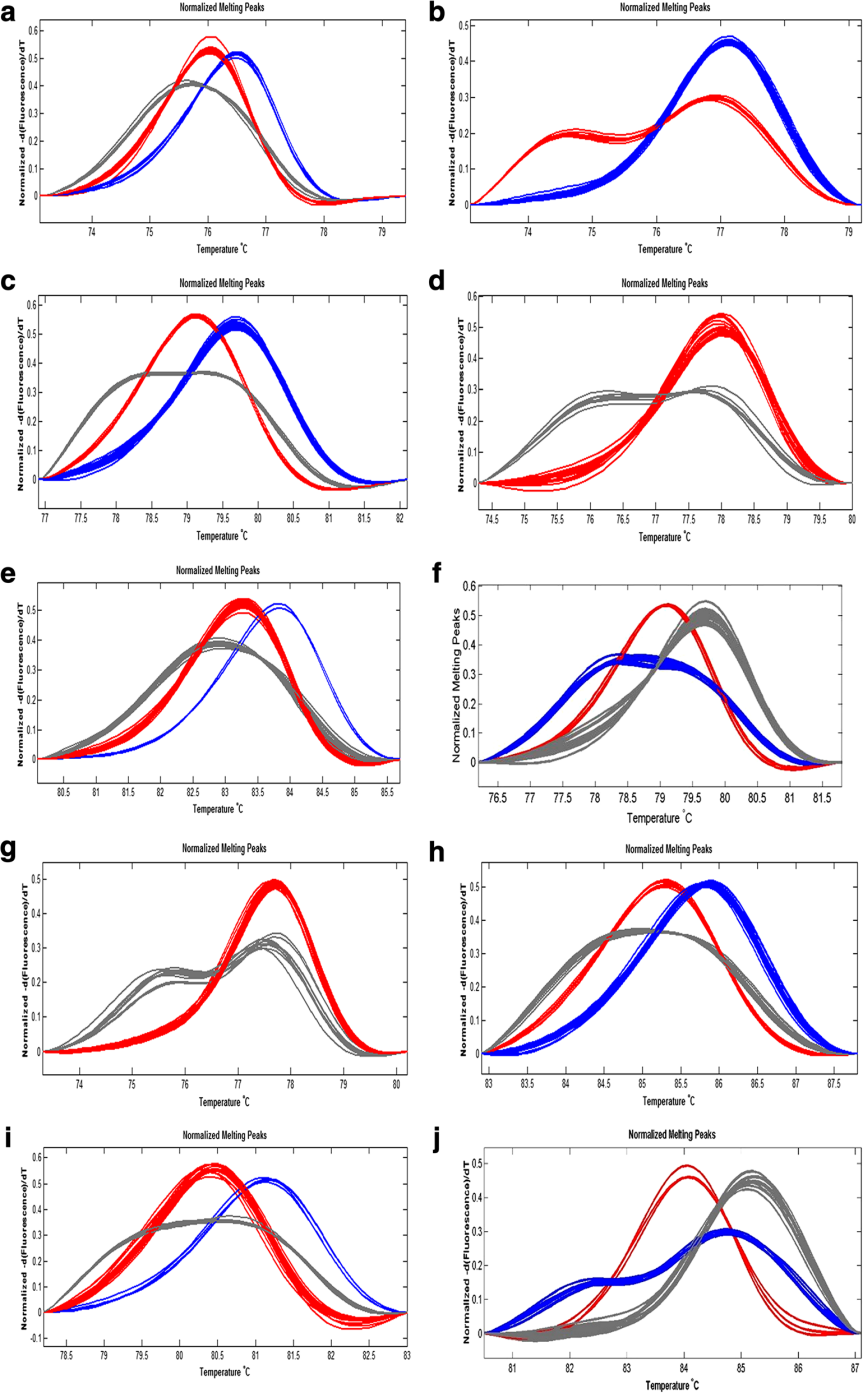
High-resolution standard melting curves of the haplotype analysis. **a** rs1024161 melting curve. Blue curve = CC genotype; red curves = CT genotype; grey curves = TT genotype. **b** rs5742909 melting curve. **c** rs231775 melting curve. Blue curve = GG genotype; red curve = AG genotype; grey curve = AA genotype. **d** rs231777 melting curve. Grey curve = CT genotype; red curve = CC genotype. **e** rs231779 melting curve. Blue curve = CC genotype; red curve = CT genotype; grey curve = TT genotype. **f** rs3087243 melting curve. Grey curve = GG genotype; red curve = AA genotype; blue curve = AG genotype. **g** rs11571319 melting curve. Grey curve = CT genotype; red curve = CC genotype. **h** rs11571302. Grey curve = CA genotype; red curve = AA genotype; red curve = CC genotype. **i** rs11571297 melting curve. Grey curve = GA genotype; red curve = AA genotype; blue curve = GG genotype **j** rs1883832 melting curve. Grey curve = CC genotype; red curve = TT genotype; blue curve = CT genotype

Genotyping results of the 10 SNPs are shown in Fig. 1a-j.

### Correlation analysis between *CTLA-4* and *CD40* polymorphism and GD susceptibility

Rs1024161, rs5742909, rs231775, rs231777, rs231779, rs3087243, rs11571319. For the 260 GD patients from the case group and 248 volunteers from the control group, under the conditions of *OR* = 1.5 and α = 0.05, all polymorphic foci had sufficient statistical power to refute the null hypothesis. The Hardy-Weinberg equilibrium test showed that the SNPs rs11571302 and rs11571297 did not match the Hardy-Weinberg equilibrium, and thus they were excluded. All other SNP foci were of good group presentation (*p* > 0.01). After age calibration, the Logistical regression analysis showed that the allelic frequencies of rs231775, rs3087243 and rs1883832 were significantly different between the normal control group and the case group (*P* < 0.05); while the distribution differences of the allelic frequencies of rs231775, rs3087243 and rs1883832 were also significantly different between the normal control group and the case group (*P* < 0.05; Tables 3 and 4).

**Table 3:** Correlation analysis between SNP allele and GD susceptibility

SNP		GD group	Control group	*P* value		*OR*(95% *CI*)	
				*χ2*	Logistic	*χ2*	Logistic
rs1024161	C	158	160	0.520	0.924	0.917	0.987
	T	362	336			0.703–1.195	0.749–1.300
rs5742909	T	52	64	0.146	0.149	0.750	0.744
	C	468	432			0.509–1.106	0.498–1.112
rs231775	G	389	328	0.002	0.001	1.521	1.593
	A	131	168			1.159–1.996	1.202–2.112
rs231777	C	462	436	0.639	0.619	1.096	1.106
	T	58	60			0.747–1.609	0.744–1.645
rs231779	T	378	360	0.968	0.987	1.006	1.002
	C	142	136			0.763–1.325	0.752–1.336
rs3087243	A	140	187	< 0.001	0.007	0.609	0.686
	G	380	309			0.467–0.794	0.520–0.903
rs11571319	T	54	60	0.388	0.562	0.842	0.887
	C	466	436			0.570–1.244	0.592–1.329
rs1883832	T	234	279	< 0.001	< 0.001	0.636	0.610
	C	286	217			0.497–0.815	0.472–0.790

Note: *OR* mutated allele frequency/wild allele frequency

**Table 4:** Correlation analysis between SNP genotype and GD susceptibility

SNP	Genotype	GD group	Control	*P* value	
				*χ2*	Logistic
rs1024161	TT/TC/CC	120/122/18	120/96/32	0.034	0.162
rs5742909	TT/TC/CC	0/52/208	0/64/184	0.119	0.122
rs231775	AA/AG/GG	15/101/144	32/104/112	0.007	0.006
rs231777	TT/TC/CC	0/58/202	0/60/188	0.615	0.593
rs231779	TT/TC/CC	138/102/20	136/88/24	0.569	0.898
rs3087243	AA/AG/GG	20/100/140	43/101/104	0.001	0.033
rs11571319	TT/TC/CC	0/54/206	0/60/188	0.355	0.534
rs1883832	TT/TC/CC	58/118/84	87/105/56	0.003	0.001

### Haplotype analysis for the correlation between *CTLA-4* and GD susceptibility

SNPs rs1024161, rs5742909, rs231775, rs231777, rs231779, rs3087243, and rs11571319 were defined as Site 1–7, respectively. Observations showed a result of *D’* < 50% and *r*^*2*^ < 0.3 among each SNP of the enrolled seven SNPs, indicating no linkage disequilibrium (LD) among these SNPs (i.e., linkage equilibrium was attained; see Table 5 for the results of the LD analysis).

**Table 5:** Linkage disequilibrium test of the commonly found *CTLA-4* SNPs in the Chinese Han population of Western Guangdong province

D’:	Site2	Site3	Site4	Site5	Site6	Site7
Site1	0.495	0.548	0.431	0.810	0.304	0.464
Site2	–	0.138	0.761	0.439	0.478	0.320
Site3	–	–	0.391	0.512	0.120	0.391
Site4	–	–	–	0.229	0.291	0.600
Site5	–	–	–	–	0.278	0.064
Site6	–	–	–	–	–	0.419
r2:	Site2	Site3	Site4	Site5	Site6	Site7
Site1	0.076	0.073	0.054	0.520	0.077	0.062
Site2	–	0.005	0.538	0.076	0.059	0.095
Site3	–	–	0.041	0.051	0.013	0.041
Site4	–	–	–	0.019	0.020	0.361
Site5	–	–	–	–	0.051	0.000
Site6	–	–	–	–	–	0.042

After excluding the haplotypes with a frequency lower than 0.05, six commonly found haplotypes remained, and these were named H_1_ to H_6_. The *χ*^2^ test showed significant differences of H_2_-H_6_ between both groups (*P* < 0.05), with H_3_ (CCGCCAC) and H_4_ (TCGCTGC) as the risk factors of GD susceptibility, while H_2_ (CCACCGC), H_5_ (TCACTAC) and H_6_ (TCACTGC) were described as preventive factors. H_4_ had the highest risk of susceptibility, while H_5_ had a maximal preventive effect (see Table 6).

**Table 6:** Haplotype analysis for the correlation between *CTLA-4* and GD susceptibility

	Haplotype	GD %	Control %	Fisher’s *P*	Person’s *P*	*OR* (95% *CI*)
H_1_	CCGCCAC	5.6	4.4	0.725096	0.725093	1.109 (0.624–1.969)
H_2_	CCGCCGC	2.0	6.0	0.000125	0.000124	0.263 (0.127–0.544)
H_3_	CCACCAC	7.5	1.7	0.000102	0.000101	4.056 (1.903–8.646)
H_4_	TCGCTGC	58.6	36.2	< 0.0001	< 0.0001	2.565 (1.883–3.495)
H_5_	TCACTAC	1.2	9.7	< 0.0001	< 0.0001	0.096 (0.042–0.222)
H_6_	TCACTGC	3.6	9.7	< 0.0001	< 0.0001	0.289 (0.166–0.503)

### Correlation analysis between SNP-SNP and SNP-sex interactions of *CTLA-4* and *CD40* and susceptibility to GD

**Fig. 2:**
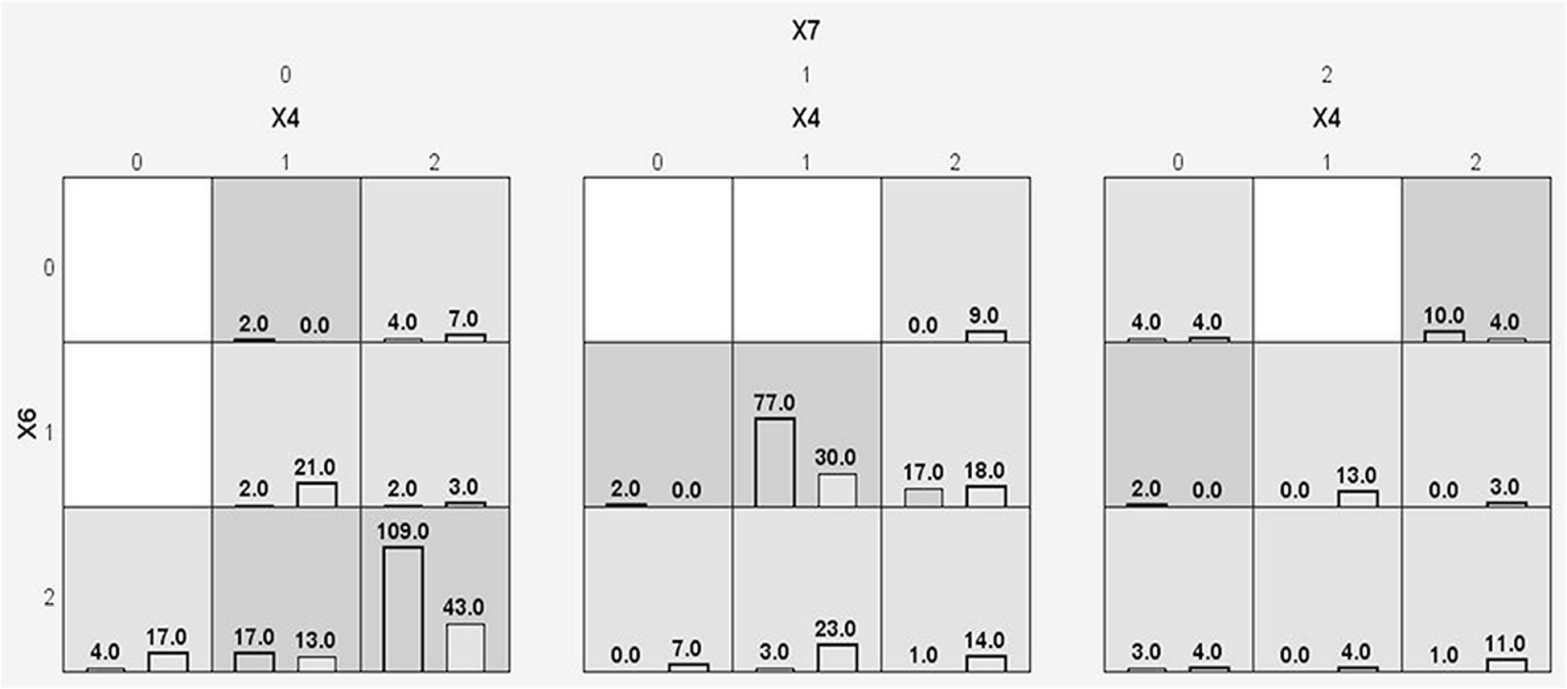
Graph model for the three-factor interaction among SNPs rs231775, rs231779 and rs3087243. The right side of the grid indicates the GD group and the left side of the grid indicates the healthy control group

Table 7 lists the study factors and their assignments. The MDR was adopted to analyze the interactions among nine influencing factors (including sex). The data are shown in Table 8. Figure 2 is the graphic model for the three-factor interactions among the SNPs of rs231775, rs231779 and rs3087243. This illustrates that the three-factor interactions among the SNPs of rs231775, rs231779 and rs3087243 exerted a greater impact upon GD susceptibility. By contrast, comparative analysis of individuals with SNPs of rs231775 (A-), rs231779 (C-) and rs3087243 (C-) showed that collaborative SNP-SNP interactions in those individuals expressing SNPs of rs231775 (GG) rs231779 (TT) and rs3087243 (GG) could increase the risk of GD by 2.5 fold (*OR* = 2.5349).

**Table 7:** Study factors and their assignments

	Study factor	Assignment
X1	sex	“male” = 1; “female” = 2
X2	rs1024161	TT = 0; CT = 1; CC = 2
X3	rs5742909	CC = 0; CT = 1; TT = 2
X4	rs231775	AA = 0; AG = 1; GG = 2
X5	rs231777	TT = 0; CT = 1; TT = 2
X6	rs231779	CC = 0; CT = 1; TT = 2
X7	rs3087243	GG = 0; GA = 1; AA = 2
X8	rs11571319	CC = 0; CT = 1; TT = 2
X9	rs1883832	CC = 0; CT = 1; TT = 2
	Outcome (Y)	GD = 1; healthy volunteer = 0

**Table 8:** Multivariate interaction model for GD susceptibility based on MDR analysis

Model	Bal.Acc.CV Testing	CV. Consistency	*P* value
X1	0.617	10/10	0.0678
X6*X7	0.7015	10/10	0.0013
X4*X6*X7	0.7055	8/10	0.0023

## Discussion

The aim of this study was to perform correlation analyses between GD and *CTLA-4* and *CD40* haplotypes to understand more about the complex genetic susceptibility of GD. In addition, we have undertaken an in-depth analysis of the interactions between *CTLA-4* and *CD40* genes in cases of GD as compared to a healthy control group. The results show that *CTLA-4* and *CD40* are associated with GD incidence in a Chinese Han population and that interactions among rs231775, rs231779 and rs3087243 SNPs significantly increased the susceptibility to GD.

Candidate gene analysis and GWAS have both confirmed that the following genes or regions are susceptible to GD: *HLA*, *CTLA-4*, *CD40, PTPN-22*, *FCRL3*, *5q31-q33*, *TSHR* and *TG* [4, 8, 14–20]. Except for *HLA*, *CTLA-4* and *CD40* are apparently the most popular susceptibility genes in autoimmune diseases. Meta-analysis has confirmed correlations between the *CD40*–1C/T(rs1883832), +49A/G(rs231775) and CT60(rs3087243) polymorphisms and the risk of GD [21, 22]. In this study, our results showed that *CD40*–1C/T(rs1883832), +49A/G(rs231775) and CT60(rs3087243) polymorphisms were associated with GD risk, but no such correlations were observed in other commonly seen SNPs. The mutant G allelic frequency of rs231775 was a risk factor of GD susceptibility, while the mutant A and T alleles of rs3087243 and rs1883832 were preventive factors. GWAS analysis suggests that SNP rs1024161 is the only *CTLA-4* SNP that shows an independent and powerful correlation with GD; however, our study indicated that rs1024161 might be irrelevant to GD occurrence in the Chinese Han population of Western Guangdong province. However, statistical false negative outcomes that might have been caused by the small sample size could not be ruled out.

GD is a complex disease that is triggered by multiple genes and multiple factors. It is inferred by GWAS that there are over 20 genes that are correlated with GD susceptibility; however, all the currently discovered variations can only explain about 20% of the causes of genetic susceptibility, and some of them still lack adequate reproducibility in other populations. These facts indicate that there may be more genetic variation that is correlated to GD susceptibility and that need to be screened (including most of the rare variants and copy number variations). In addition, further elucidations of the potential mechanisms and functions of the variations, including gene-gene and gene-environment interactions might improve the genetic interpretation of GD susceptibility.

Leakage disequilibrium (LD) analysis was adopted to assess the seven SNPs of the normal Chinese Han population in Western Guangdong province. The results showed *D’* < 50% and *r*^*2*^ < 0.3 among each SNP, which indicated no LD among these SNPs (i.e., linkage equilibrium was reached). Since there is a high possibility of free gene recombination and allocation, while there is a low possibility of random drift and selection among commonly seen *CTLA-4* SNPs in the Chinese Han population of Western Guangdong province, we selected all seven SNPs described above (i.e., rs1024161, rs5742909, rs231775, rs231777, rs231779, rs3087243, rs11571319) so that we could conduct a case-control haplotypic analysis. After excluding haplotypes with a frequency < 0.05, six haplotypes remained, and the TCGCTGC haplotype was the risk haplotype that displayed the highest frequency, while the TCACTAC haplotype was the preventive haplotype. Risk of GD in individuals with the TCGCTGC haplotype was increased by 2.6 fold while the risk in those with a TCACTAC haplotype was increased by 0.096 fold. Gu et al. [5] analyzed the ACACC and ACGCT haplotypes that were composed of five SNPs in *CTLA-4* (i.e., rs4553808, rs5472909, rs231775, rs231777 and rs231779), and discovered that the ACGCT haplotype increased GD risk by 1.6 fold, while the ACACC haplotype reduced the risk by 1.26 fold. Therefore, haplotypes not only break through the limitations of individual SNP analysis, but they can also reduce the difference between studies.

*CTLA-4* is undoubtedly one of the susceptibility genes of GD occurrence, but its variations that correlate to the risk of GD differ greatly in different populations. Moreover, in studies that have been conducted on human haplotypes, all known SNPs of a particular gene can be studied in a holistic way, and it is possible to detect tagged SNPs to certain regions of a haplotype, which greatly improves research efficiency, and solves the problem of low testing efficiency that is caused by individual SNP testing, which thus enables us to derive an overall correlation between *CTLA-4* and GD [23].

We adopted the MDR model to assess the interactions among nine factors (including sex) and found that three-factor interactions among the SNPs of rs231775, rs231779 and rs3087243 had the greatest impact upon GD susceptibility. Interactions between rs231779 and rs3087243 exhibited a strong antagonistic effect, but this effect was alleviated after adding rs231775. Both *CTLA-4* and *CD40* are immunomodulatory genes, but we did not find interactions of *CTLA-4* and *CD40* that were associated with the risk of GD via MDR analysis. Similarly, Yang et al. [10] also found that there was no multiplied interaction between *CD40* C/T (− 1) and *CTLA-4* A/G (49) SNPs associated with GD susceptibility. However, another study has shown that there may be a combined and additive effect between the CD40–1C > T and CTLA-4-6230G > A polymorphisms with the development of GD [13]. Other studies have shown that interactions between *CTLA-4* and other genes also increased the risk of GD. For example, interactions between *HLA*-DPB1*0501 and the *CTLA4*-CT60 polymorphism conferred a 9.99 fold GD risk [24] and synergistic interactions may exist between SNP rs231779 in *CTLA-4* and rs2069550 in *TG* [5]. We therefore infer that the mechanisms of *CTLA-4* participating in GD pathogenesis are more complicated than we had originally appreciated, and might involve mechanisms of both intra- and extragenic interactions.

This study has some limitations, including the small sample size that was mentioned above that may have given false negative outcomes. Since we could not determine the major and minor effects of interactions from each level by MDR analysis, we could not differentiate the contributions made by each SNP (i.e., rs231775, rs231779 and rs3087243) to the risk of GD based on interaction analysis. The results only represent statistically related or unrelated, and our results require further biology. In-depth research in the sense to confirm or correct. All in all, our research is now more precisely a cue for future research than a powerful evidence to clarify the etiology of GD genetics.

## Conclusion

In conclusion, we performed a retrospective case control analysis in the Chinese Han population of the Western Guangdong province (260 GD patients and 248 healthy volunteers), and showed that *CTLA-4* and *CD40* were correlated with susceptibility to GD. The mutant G allelic frequency of rs231775 was a risk factor of GD susceptibility, while the mutant A and T alleles of SNPs rs3087243 (CT60) and rs1883832 (-1C/T polymorphism) in *CD40* were preventive factors*.* Individuals with a *CTLA-4* TCGCTGC haplotype (i.e., rs1024161, rs5742909, rs231775, rs231777, rs231779, rs3087243 and rs11571319) were susceptible to GD, while those with a TCACTAC haplotype were not. Synergistic interactions of the *CTLA-4* SNPs rs231775, rs231779 and rs3087243 significantly increased the risk of GD.

## Abbreviations

GD: Graves’ disease
GWAS: Genome-wide association studies
LD: Linkage disequilibrium
SNP: Single-nucleotide polymorphism
